# The Association of Calcium Signaling Pathway Gene Variants, Bone Mineral Density and Mild Cognitive Impairment in Elderly People

**DOI:** 10.3390/genes14040828

**Published:** 2023-03-30

**Authors:** Jiesong Zhang, Xueyan Wang, Haiping Duan, Chen Chen, Zhonghai Lu, Dongfeng Zhang, Suyun Li

**Affiliations:** 1 Department of Epidemiology and Health Statistics, School of Public Health, Qingdao University, Qingdao 266012, China; 2 Qingdao Municipal Center for Disease Control and Prevention, Qingdao 266033, China

**Keywords:** calcium signaling pathway, mild cognitive impairment, bone mineral density, polygenic risk scores, interaction effect

## Abstract

The association of calcium signaling pathway gene variants, bone mineral density (BMD) and mild cognitive impairment (MCI) is poorly understood so far. A total of 878 participants from Qingdao city were recruited in this study. According to the candidate gene selection method, 58 single nucleotide polymorphisms (SNPs) in eight calcium signaling genes were selected. The association between gene polymorphisms and MCI was revealed by using multiple genetic models. Polygenic risk scores (PRS) were used to summarize the effects of the whole gene. Logistic regression was used to analyze the association between each PRS and MCI. The multiplicative interaction term in the regression models was used to estimate the interaction effects between the PRS and BMD. We observed significant associations of rs6877893 (NR3C1), rs6448456 (CCKAR), and rs723672 (CACNA1C) polymorphisms with MCI. The PRSs of NR3C1 (OR = 4.012, 95% CI = 1.722–9.347, *p* < 0.001), PRKCA (OR = 1.414, 95% CI = 1.083–1.845, *p* = 0.011) and TRPM1 (OR = 3.253, 95% CI = 1.116–9.484, *p* = 0.031) were associated with an increased risk of developing MCI, and the PRS of total genes (OR = 0.330, 95% CI = 0.224–0.485, *p* < 0.001) was associated with a decreased risk of developing MCI. In interaction effect analysis, the interaction effect of PRKCA and BMD was significant. Genetic variations of the calcium signaling pathway were associated with MCI in older people. There was an interaction effect between PRKCA gene variants and BMD on MCI.

## 1. Introduction

Mild cognitive impairment (MCI), a complex status of cognitive decline, is generally considered as the intermediate stage between the changes in normal cognitive aging and dementia [1]. It is reported that MCI may occur during the whole process of normal aging, though it is more commonly noticed in the elderly [2,3]. According to a recent cross-sectional study, the overall population of individuals with MCI and dementia is estimated to account for more than one in five adults aged 60 years or older in China [4]. There is currently no cure for dementia, so it is necessary to target MCI interventions to prevent the onset of dementia [3,5]. Recently, a review provided evidence that genetic variants can predict aging-related cognitive impairment, which supported the potential role of genetic factors [6]. Differences in individual genetic susceptibility are associated with cognitive status in later life, so further investigation of the relationship between genetic variants and cognitive function is warranted.

In recent years, single nucleotide polymorphisms (SNPs) linked to cognitive function have been identified by genome-wide association studies (GWAS) and candidate gene studies [7,8]. In our previous GWAS of cognitive function in middle and old-aged adults of 139 twins in northern China, many SNPs associated with cognitive function were discovered to be enriched in the calcium signaling pathway [9]. Other reported studies also showed that the calcium signaling pathway was a vital component of the mechanisms responsible for information processing and the formation of memory and cognition [10,11]. Moreover, a study has shown significant and consistent enrichment for genes that constituted the calcium signaling pathway [12]. Calcium serves as a second messenger regulating synaptic plasticity, with most of its work focused on the hippocampus, which is a crucial brain region engaged in learning and memory [13]. Moreover, a calcium hypothesis of Alzheimer’s disease (AD) held that calcium can affect neuronal function in multiple aspects such as temporal, concentration and environmental factors, which meant that abnormal gene variants may induce both the progressive decline in memory and the increase in neuronal cell apoptosis, resulting in MCI and even AD [14].

In the current genetic studies of cognitive function, most studies only investigated a single gene or single SNP of one gene in the calcium signaling pathway. However, less has been mentioned about the overall effect of multiple SNPs or genes on cognitive functions. Polygenic risk score (PRS) is an algorithm that combines information on the variation of all SNPs in one gene, reflecting the overall variation in that gene, which can increase the test power and achieve higher detection efficiency [15,16]. Thus, in the present study, we will aim to investigate the association between the calcium signaling pathway PRS and MCI in the elderly after studying the polymorphism of a single locus.

Though the etiology of MCI is inconclusive, environmental and lifestyle factors, such as physical activity [17,18], sleep [19], depression, anxiety and loneliness [20,21], have been mentioned to play a crucial role in its risk, onset and progression [22,23]. Moreover, epidemiological evidence suggests that people with osteoporosis have a higher incidence of cognitive impairment than the general population, especially in executive function, processing speed and verbal memory [24,25,26,27]. Hence, continued elucidation of the interaction between genetic variation in calcium signaling pathways and bone mineral density (BMD) is expected to facilitate the development of effective strategies for the prevention of MCI.

Therefore, in this study, we mainly aimed to verify the associations of genetic variants of the calcium signaling pathway with MCI and explore whether there were interaction effects between BMD and genetic variants in each gene.

## 2. Materials and Methods

### 2.1. Subjects and Procedure

In this study, individuals aged 60 years and above who were permanent residents of Qingdao were included in the study, after which, patients with severe physical or mental illnesses and individuals who were uncooperative in completing the survey were excluded. A face-to-face questionnaire survey was conducted with the community participants. To enhance the research quality of the investigation, all investigators were rigorously trained to fully understand the study, be familiar with the content of the questionnaire and acquire proficient and consistent questioning skills before the investigation. The self-reported questionnaire included demographic information, behavior and lifestyle such as smoking and alcohol consumption, and cognitive function test using the Montreal Cognitive Assessment (MOCA). Moreover, physical measurements and blood samples were also collected. The study was conducted in accordance with the Declaration of Helsinki, and the study proposal was endorsed by the Ethics Committee of Qingdao University Medical College. Signed consents were obtained from all participants after the purpose of this study was fully explained.

### 2.2. Measures

#### 2.2.1. Cognitive Function

Cognitive function was measured using the MOCA test, which included eight cognitive subtests: visual–spatial function, executive function, short-term memory, delayed memory, language, attention, abstraction, calculation, and orientation. The MOCA test was widely seen as a brief cognitive screening instrument to recognize mild cognitive impairment (MCI) and early dementia [28]. Scores ranged from 0 to 30, with an extra point added if the participant had ≤12 years of schooling, with higher scores indicating better cognitive behavior, and a score of <26 was identified as the optimum cutoff point for a definition of cognitive impairment [28]. Therefore, individuals with 26 points and greater were considered in a normal cognitive state and others were considered as MCI in our study. The total scale showed good internal consistency reliability in this study (Cronbach’s α = 0.707).

#### 2.2.2. Bone Mineral Density

The bone mineral density was measured by professionals using a dual-energy X-ray absorptiometry (DXA) bone densitometer [29].

### 2.3. Gene Selection

Based on the previous GWAS of cognitive function in middle- and old-aged adults of 139 pairs of dizygotic twins [9], a list of significant genes (*p* < 0.05) was submitted to the GSEA (gene set enrichment analysis) website (https://www.gsea-msigdb.org/gsea/msigdb/index.jsp, accessed on 29 January 2023) to find pathways that were over-represented in the list of significant genes underlying cognitive function. Finally, eight genes comprised in the calcium signaling pathway were chosen by using the candidate gene selection method, including the autophagy-related 12 gene (ATG12), the BAF chromatin remodeling complex subunit gene (BCL11B), the calcium voltage-gated channel subunit alpha1 C gene (CACNA1C), the cholecystokinin A receptor gene (CCKAR), nuclear receptor subfamily 3 group C member 1 gene (NR3C1), protein kinase C α gene (PRKCA), the Rac family small GTPase 1 gene (RAC1) and the transient receptor potential cation channel subfamily M member 1 gene (TRPM1).

### 2.4. SNPs Selection and Imputation

A total of 58 SNPs of the above eight genes were chosen by using the candidate gene selection method, and the principal criteria were as follows: (1) The tag-SNPs identified with a minor allele frequency (MAF) > 0.05 and the minimum linkage disequilibrium correlation (r2) > 0.8 in the database of the Chinese Han Beijing (CHB) population data of HapMap (HapMap Data Rel 27 PhaseII + III) using Haploview software 4.2. (2) The valid function SNPs were selected from the CHB population data of the dbSNP database (https://www.ncbi.nlm.nih.gov/snp/, accessed on 29 January 2023) and the 1000 Genomes Project, after which, the selected SNPs were used for functional prediction (http://snpinfo.niehs.nih.gov/, accessed on 29 January 2023) and for linkage disequilibrium analysis by an ensemble (http://asia.ensembl.org/Homo_sapiens/Tools/LD?db=core, accessed on 29 January 2023). (3) The SNPs previously reported in the literature that were associated with cognitive function were selected [30,31].

Leukocytes were isolated within 2 h of blood collection from a pre-prepared tube stored with an EDTA anticoagulant storage [32]. A DNA extraction kit (BioTeke Corporation, Beijing, China) was used to isolate and purify genomic deoxyribonucleic acid (DNA) from the peripheral blood of each individual. According to the sequence information of the SNPs, PCR and single-base extension primers were designed using Assay Design 3.1. SpectroCHIP was obtained after PCR amplification, product alkaline phosphatase treatment, single-base extension reaction, resin purification, and microarray spotting. MassArray (Sequenom Inc., San Diego, CA, USA) used allele-specific matrix-assisted laser desorption/ionization time-of-flight mass spectrometry (MALDI- TOF) to genotype all data. The high-frequency allele was deemed as the major allele and the low-frequency allele as the minor allele. Genotype imputation was performed using the multiple imputation by chained equations (MICE) algorithm, which was a flexible and practical method to deal with missing data [33].

### 2.5. Calculation of the PRS

To increase our research effectiveness, the effect of each gene was studied, and the weighted PRS for each gene was computed as the total of the weights of the estimated genetic effect sizes corresponding to each allele [15,34]. Based on the additive genetic model, linear regression was used to investigate the relationship between cognitive function and the integrated effects of SNPs of each gene. In this study, each SNP was considered as an independent variable and MOCA scores were deemed to be the dependent variable. The PRS was calculated using the following formula:

PRS = w_1_ × SNP_1_ + w_2_ × SNP_2_ + ... + w_k_ × SNP_k_

where SNP_i_ is the number of risk alleles, w_i_ is the weight of each SNP and k is the number of SNPs used (i.e., k = 58).

### 2.6. Statistical Analysis

The statistical analyses were performed with STATA/MP version 15.0 (Stata Corporation, College Station, TX, USA.) and a 2-sided *p* < 0.05 was considered as the criterion for statistical significance. The demographic characteristics across the different groups were described as frequency (proportion) for classified variables and as mean ± standard deviation (SD) for continuous variables, and independent *t*-tests and Pearson’s Chi-squared test was used to compare the differences in cognitive function for the different variables. The Chi-square test was used to determine whether the genotype frequency distribution of each SNP followed the Hardy–Weinberg equilibrium. In the allele, dominant, recessive, homozygote and heterozygote models, the logistic regression was used to investigate the relationships between each SNP and MCI, respectively. Quality control was performed prior to imputation and subjects with > 10% missing genotype data were excluded. After multiple imputation by chained equations, and association analyses, the PRS of each gene and total PRS were obtained for subsequent analyses. Then, the logistic regression was carried out to investigate odds ratios (OR) for each PRS and MCI in crude models and multivariate-adjusted models (adjusted for age, gender, education, smoking status and alcohol drinking status), respectively. Finally, the interaction effects between the PRS and BMD on cognitive function were estimated by including the respective multiplicative interaction term in the logistic regression models, and marginal effects were plotted to provide a clear, visual illustration of the underlying interaction effects.

## 3. Results

### 3.1. Sample Characteristics

The demographic characteristics of 571 cognitively impaired and 307 cognitively normal individuals were described in Table 1. Compared with the normal cognitive status group, the mild cognitive impairment group was inclined to have a significantly higher age (t = −5.90, *p* < 0.001) and a significantly lower BMD (t = 5.31, *p* < 0.001). Distributions of gender (χ^2^ = 36.19, *p* < 0.001), education (χ^2^ = 74.79, *p* < 0.001), smoking status (χ^2^ = 27.33, *p* < 0.001) and alcohol drinking status (χ^2^ = 19.86, *p* < 0.001) between the mild cognitive impairment and normal cognitive status groups were significantly different and the mild cognitive impairment group typically had more women, lower education levels, and lower proportions of smoking and drinking status.

### 3.2. Results of the Relationship between Gene Polymorphism and MCI

Detailed information on 58 SNPs for the eight genes in this study was given in Supplementary Table S1. Alleles of all 58 SNPs were consistent with the law of genetic balance after the Hardy–Weinberg equilibrium test (*p* > 0.05). In the analysis of the relationship between SNP and cognitive function after adjusting all covariates, we identified three SNPs (rs6877893, rs64458456 and rs723672) that were associated with MCI in Table 2.

For rs6877893 polymorphism, individuals with mutated allele AA genotype had a decreased risk of cognitive impairment susceptibility compared to individuals with allele AA or AG genotypes (OR = 0.52, 95%CI: 0.27–0.99, *p* = 0.045). No significant association was detected between rs6877893 polymorphism and MCI in the allele model (A vs. G) (OR = 1.02, 95%CI: 0.81–1.92, *p* = 0.881), the dominant model (AA vs. AG + GG) (OR = 1.14, 95%CI: 0.84–1.55, *p* = 0.392), the homozygote model (AA vs. GG) (OR = 0.57, 95%CI: 0.29–1.12, *p* = 0.101) and the heterozygote model (AA vs. AG) (OR = 1.29, 95%CI: 0.93–1.78, *p* = 0.129).

For rs6448456 polymorphism, there was a significant association with a decreased risk of MCI in the recessive model (GG + GC vs. CC) (OR = 0.32, 95%CI: 0.11–0.90, *p* = 0.031) and the homozygote model (GG vs. CC) (OR = 0.34, 95%CI: 0.12–0.98, *p* = 0.045). In addition, the results displayed no significant association in the allele model (G vs. C) (OR = 1.07, 95%CI: 0.81–1.43, *p* = 0.628), the dominant model (GG vs. GC + CC) (OR = 1.15, 95%CI: 0.82–1.63, *p* = 0.419) and the heterozygote model (GG vs. GC) (OR = 1.29, 95%CI: 0.90–1.86, *p* = 0.167).

For rs723672 polymorphism, individuals with the mutated allele TC genotype had an increased risk of MCI susceptibility compared to individuals with the allele TT genotype (TT vs. TC) (OR = 1.40, 95%CI: 1.01–1.93, *p* = 0.042). However, there was no significant association between rs723672 polymorphism and MCI in the allele model (T vs. C) (OR = 1.12, 95%CI: 0.89–1.42, *p* = 0.347), the dominant model (TT vs. TC + CC) (OR = 1.30, 95%CI: 0.96–1.77, *p* = 0.090), the recessive model (TT + TC vs. CC) (OR = 0.72, 95%CI: 0.37–1.41, *p* = 0.344) and the homozygote model (TT vs. CC) (OR = 0.84, 95%CI: 0.43–1.64, *p* = 0.615).

### 3.3. Results of the Relationship between PRS and MCI

Table 3 showed the association between the PRS of each gene and MCI by applying logistic regression. In the crude model, the univariate logistic regression indicated that the ATG12, NR3C1, PRKCA and TRPM1 genes were risk factors for MCI, indicating that a higher ATG12, NR3C1, PRKCA and TRPM1 gene score was significantly associated with an increased risk of MCI. At the same time, the CACNA1C and CCKAR genes were protective factors for MCI, manifesting that higher PRSs of CACNA1C and CCKAR were associated with a decreased risk of MCI. In the multivariate logistic regression analysis, after adjusting for age, gender, education, smoking status and alcohol drinking status, the results remained significant (NR3C1, PRKCA and TRPM1 gene score). The PRS of the total gene in the calcium signaling pathway for all SNPs was also significantly associated with cognitive impairment (*p* < 0.001; multivariate-adjusted OR: 0.330, 95%CI: 0.224,0.485).

### 3.4. Sensitivity Analysis

Considering the potential influence of gender on cognition, estrogens may play a relevant role in calcium metabolism, so we performed a subgroup analysis of gender (Table 4). The association between the PRS of the NR3C1, PRKCA, and TRPM1 genes and MCI was significant in women but not significant in men. However, after including the PRS with the gender interaction term in the regression, the interaction effect between gender and each PRS was not significant.

### 3.5. Interaction Effect Analysis between PRS and Bone Mineral Density

**Figure 1:**
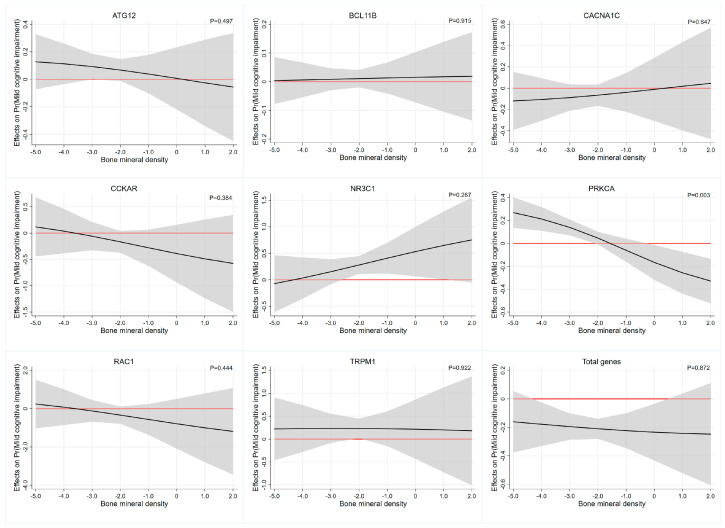
The average marginal effect of the PRS across the BMD range. Each subgraph represents the interaction effect analysis of the association between each PRS and BMD on cognitive function. Black lines represent the marginal effect of the PRS estimated across the BMD range and the gray regions depict 95% confidence intervals. Red lines denote a marginal effect of zero. Abbreviations: ATG12, autophagy-related 12 gene; BCL11B, BAF chromatin remodeling complex subunit BCL11B; CACNA1C, calcium voltage-gated channel subunit alpha1 C gene; CCKAR, cholecystokinin A receptor gene; NR3C1, nuclear receptor subfamily 3 group C member 1 gene; PRKCA, protein kinase C α gene; RAC1, Rac family small GTPase 1 gene; TRPM1, transient receptor potential cation channel subfamily M member 1 gene.

Interaction effect analysis of the association between each PRS and BMD on cognitive function was described in Table 5. Based on the multiplicative interaction model, uncorrected estimates revealed that BMD levels moderated the association between PRS of PRKCA and cognitive function (OR = 0.638, 95%CI: 0.463–0.879, *p*-interaction= 0.006). After adjusting for age, gender, education, smoking status and alcohol drinking status, we also revealed that the PRS of PRKCA had significant interactions with BMD (OR = 0.593,95%CI: 0.418–0.841, *p*-interaction= 0.003) and the effects were most evident at the higher and lower ends of the distribution of BMD levels. Specifically, the value of the marginal effect of BMD decreases gradually as the PRS of PRKCA and the marginal effect of BMD is not significant beyond the *p* < 0.05 significance threshold between the BMD, approximately −2.0 to 0 (Table 4 and Figure 1). The interaction results of BMD with other PRSs on cognitive function were similar, but not significant. This was more clearly illustrated in Figure 1, where the widening of the confidence intervals at the heads and tails of the PRS distribution were associated with imprecise estimates of the association.

## 4. Discussion

In this study, multiple genetic models were employed to examine the association between individual SNPs and cognitive function in calcium signaling pathways. It was found that rs6877893 (NR3C1), rs6448456 (CCKAR), and rs723672 (CACNA1C) polymorphisms were significantly correlated with MCI. We employed a polygenic risk score algorithm to verify the candidate genes in the calcium signaling pathway in order to further account for genetic elements related to cognitive function in the elderly in northern China. Specifically, we found that the NR3C1, PRKCA and TRPM1 gene variants were more likely to be risk factors for MCI, and after adjusting for confounding variables, the total PRSs of all the SNPs in the calcium signaling pathway will decrease the risk of developing MCI. Based on the multiplicative interaction model, we found that the PRS of PRKCA had a significant interaction effect with BMD after adjusting for all covariates. Our findings substantiated the former GWAS results that the calcium signaling pathway gene variants were connected with cognitive function. On the other hand, we also found that a gene of the calcium signaling pathway may interact with BMD to affect cognitive function.

In recent years, a growing number of studies have confirmed the relationship between SNP and cognition in calcium signaling pathways. A meta-analysis of SNP association results from both cohorts revealed that rs6877893 (NR3C1) nominally associated with reasoning influenced perceptual performance in cognitively healthy individuals [35]. Tachikawa et al. found that the rs6448456 locus in CCKAR was associated with cognition in the Spanish population [36]. A case–control study by Porcelli, S. et al. found that the rs723672 locus in the CACNA1C gene was related to the improvement of symptoms of schizophrenia, which was characterized by structural abnormalities in the brain as well as cognitive impairments, indicating that this locus might play a role in regulating cognitive state [37,38]. Therefore, based on the previous literature data, we studied the polymorphism of a single locus and found that in the polymorphism of rs6877893, the susceptibility to cognitive impairment of individuals with AA genotype mutation was lower than that of individuals with AA or AG genotype mutation. In rs6448456 polymorphism, the recessive model and the homozygous model are significantly related to the risk reduction in cognitive function. For rs723672 polymorphism, the correlation between rs723672 polymorphism and cognitive function susceptibility is only significant in the heterozygote model. The results suggest that the three polymorphisms, related to processes of calcium metabolism, are associated with possible areas of the nervous system, which may manifest as MCI.

The NR3C1 gene is located on chromosome 5q31.3 whose main function is to encode for the glucocorticoid receptor (GR) and the evidence has shown cortisol levels in humans could be affected by this gene [39]. In the meantime, the influences on cognitive function are notably modulated by the binding of cortisol to the lower affinity GR [39,40]. To be precise, GR can impact learning and memory function specifically by affecting synaptic transmission effectiveness, neural structure integration, and the formation of LTP, which was the foundation of memory formation [41]. Keller et al. researched the connection between Hypothalamic–pituitary–adrenal axis activity, cortisol, clinical emotional symptoms, and genetic variants and cognition, and proposed that genetic variants of NR3C1 were involved in attention and working memory [42]. In our study, we verified the association of PRS of NR3C1 with cognitive function in the elderly in northern China.

Protein kinase C (PKC), a family of phospholipid-dependent serine/threonine protein kinases, forms a comprehensive signaling network in the brain and has been confirmed to be involved in different cellular signaling pathways [43]. For instance, PKC can promote the release of several growth factors that promote neural stem cells to produce new neurons that integrate into hippocampal circuits, which are widely associated with memory and cognitive performance [44]. To date, an animal experiment has revealed that PKC isoforms such as PKCα (PRKCA) were closely correlated with pathological damage in AD [45]. Moreover, regarding the memory process, PKC isozymes, particularly PKCα, shared essential roles in signaling pathways, and studies have verified SNPs (rs4790904, rs8074995) in the gene encoding PRKCA were found to be markedly associated with memory [46,47]. In this study, the PRS of PRKCA was strongly associated with cognitive impairment, which was favorably consistent with previous studies and animal experiments. What is noteworthy is that previous studies have indicated the causal relationship between cognitive impairment and low BMD [24,25,26,27]. Further, in our study, the different levels of PRKCA gene variants with the modulation of BMD may have affected the cognitive outcomes differently.

In recent years, relatively little research has been published on the TRPM1 gene and cognitive function. The existing papers pointed out that TRPM1 gene mutations commonly occur in 15q13.3 microdeletion syndromes and that deletion of the TRPM1 gene products has been considered as a possible mechanism contributing to severe visual impairment [48]. Recently, a mice experiment proved the cognitive impairment observed in the 15q13.3 microdeletion syndrome phenotype, demonstrating that TRPM1 gene mutations may be associated with impaired cognitive function [49]. In this study, we came to the same conclusion as a population study, but the exact biological mechanism will need further study.

In this study, we performed interaction effect analysis to explore possible mechanisms underlying the association between the PRS of the calcium signaling pathway and cognitive function. Furthermore, several potential explanations for the association between BMD and cognitive impairment are as follows: First, bone mass loss may cause an increase in certain inflammatory markers, such as interleukin-6 [50] and TNF-α [51], which promote the accumulation of inflammatory plaques in the brain. Second, abnormal calcium and phosphorus metabolism can induce an excessive influx of calcium and death of the neuron cells, which may aggravate the formation of senile plaques and neurofibrillary tangles in AD [52]. Besides, previous studies have certified that osteocalcin can cross the blood–brain barrier and induce the accumulation of calcium and excitatory neurotransmitters in neurons, thereby affecting cognitive function [53,54]. In the end, estrogen as a significant hormone in bone homeostasis, has been proven to affect cognitive function through diverse mechanisms [55].

As previously mentioned, a major strength of this study is that the association analysis was carried out using PRS, which can combine the impacts of many genetic variants loci with small effects. Moreover, we conducted the mediation analysis on cognitive function, which may provide another pathway to explain the effect of BMD on cognitive function.

Nevertheless, there were some potential limitations in this study. Firstly, since this is a case–control study, our results could be interfered with by selection and recall bias. Secondly, the sample size in our study was relatively small, so larger sample sizes are required to validate our study in future studies. Finally, a greater proportion of our study participants were women, who were generally less likely to have risk factors such as smoking or alcohol consumption, which may have contributed to the differences with previous studies.

In summary, this case–control study based on northern Chinese older people verified associations between the calcium signaling pathway gene and cognitive function and identified an interaction effect between PRKCA gene variants and bone mineral density on mild cognitive impairment. This study provided another way to explore the influence of genetic factors affecting cognitive function. Furthermore, future studies were expected to confirm our findings, as well as improve our results in a larger population.

## Figures and Tables

**Table 1:** The characteristics of the sample by cognitive functions.

Characteristics	Normal Cognitive Status (*n* = 307)	Mild Cognitive Impairment (*n* = 571)	*p*-Value
Age (years)	66.92 ± 5.01	69.44 ± 6.54	<0.001 ^1^
Gender (*n* (%))			<0.001 ^2^
Male	164 (53.4)	186 (32.6)	
Female	143 (46.6)	385 (67.4)	
Education (*n* (%))			<0.001 ^2^
Low	3 (1.0)	100 (17.5)	
Middle	268 (87.3)	455 (79.7)	
High	36 (11.7)	16 (2.8)	
Smoking status (*n* (%))			<0.001 ^2^
Never	169 (55.0)	414 (72.5)	
Ever	74 (24.1)	82 (14.4)	
Current	64 (20.9)	75 (13.1)	
Alcohol drinking status (*n* (%))			<0.001 ^2^
Never	169 (55.0)	393 (68.8)	
Ever	10 (3.3)	24 (4.2)	
Current	128 (41.7)	154 (27.0)	
BMD (T-value)	−2.08 ± 0.87	−2.40 ± 0.83	<0.001 ^1^

Notes: Data are presented as Mean ± SD or frequency. Education: low = less than primary school, middle = primary and middle school, high = high school or more; BMD: bone mineral density. ^1^ Student *t*-test; ^2^ Pearson’s Chi-squared test.

**Table 2:** The results of allele, dominant, recessive, homozygote and heterozygote regression models for a single SNP.

SNP	Model	Genotype ^1^	OR	95%CI		*p*-Value
				Low	Upper	
rs6877893	Allele model	A vs. G	1.02	0.81	1.29	0.881
	Dominant model	AA vs. AG + GG	1.14	0.84	1.55	0.392
	Recessive model	AA + AG vs. GG	0.52	0.27	0.99	0.045 *
	Homozygote model	AA vs. GG	0.57	0.29	1.12	0.101
	Heterozygote model	AA vs. AG	1.29	0.93	1.78	0.129
rs6448456	Allele model	G vs. C	1.07	0.81	1.43	0.628
	Dominant model	GG vs. GC + CC	1.15	0.82	1.63	0.419
	Recessive model	GG + GC vs. CC	0.32	0.11	0.90	0.031 *
	Homozygote model	GG vs. CC	0.34	0.12	0.98	0.045 *
	Heterozygote model	GG vs. GC	1.29	0.90	1.86	0.167
rs723672	Allele model	T vs. C	1.12	0.89	1.42	0.347
	Dominant model	TT vs. TC + CC	1.30	0.96	1.77	0.090
	Recessive model	TT + TC vs. CC	0.72	0.37	1.41	0.344
	Homozygote model	TT vs. CC	0.84	0.43	1.64	0.615
	Heterozygote model	TT vs. TC	1.40	1.01	1.93	0.042 *

Abbreviations: OR: odds ratio; CI: confidence interval. * Represents the significance of gene score association analysis (*p* < 0.05). ^1^ The former of the genotypes is used as the reference genotype.

**Table 3:** The relationship between PRSs of the calcium signaling pathway and MCI.

Gene	Crude Model ^1^		Multivariate-Adjusted ^2^	
	OR (95 %CI)	*p*-Vaule	OR (95 %CI)	*p*-Vaule
ATG12	1.589 (1.113–2.270)	0.011 *	1.460 (0.998–2.133)	0.051
BCL11B	1.064 (0.930–1.217)	0.370	1.052 (0.908–1.218)	0.502
CACNA1C	0.636 (0.410–0.986)	0.043 *	0.697 (0.432–1.124)	0.139
CCKAR	0.370 (0.144–0.950)	0.039 *	0.480 (0.174–1.328)	0.158
NR3C1	4.739 (2.148–10.459)	<0.001 *	4.012 (1.722–9.347)	0.001 *
PRKCA	1.511 (1.181–1.934)	0.001 *	1.414 (1.083–1.845)	0.011 *
RAC1	0.143 (0.020–1.037)	0.054	0.224 (0.027–1.862)	0.166
TRPM1	5.477 (1.966–15.261)	0.001 *	3.253 (1.116–9.484)	0.031 *
Total	0.265 (0.186–0.380)	<0.001 *	0.330 (0.224–0.485)	<0.001 *

Abbreviations: ATG12, autophagy-related 12 gene; BCL11B, BAF chromatin remodeling complex subunit BCL11B; CACNA1C, calcium voltage-gated channel subunit alpha1 C gene; CCKAR, cholecystokinin A receptor gene; NR3C1, nuclear receptor subfamily 3 group C member 1 gene; PRKCA, protein kinase C α gene; RAC1, Rac family small GTPase 1 gene; TRPM1, transient receptor potential cation channel subfamily M member 1 gene; Total: PRS for the whole SNPs; OR: odds ratio; CI: confidence interval; PRS: polygenic risk scores; MCI: mild cognitive impairment. * Represents the significance of gene score association analysis (*p* < 0.05). ^1^ Crude results. ^2^ Results adjust for age, gender, education, smoking status, and alcohol drinking status.

**Table 4:** The subgroup analysis of the relationship between PRSs of calcium signaling pathway and MCI.

Gene	Male		Female	
	OR (95 %CI)	*p*-Vaule	OR (95 %CI)	*p*-Vaule
ATG12	1.429 (0.820–2.488)	0.207	1.364 (0.796–2.336)	0.258
BCL11B	1.271 (0.997–1.620)	0.053	0.957 (0.792–1.155)	0.646
CACNA1C	0.555 (0.260–1.181)	0.126	0.819 (0.436–1.540)	0.536
CCKAR	0.589 (0.125–2.784)	0.504	0.321 (0.080–1.290)	0.109
NR3C1	2.705 (0.768–9.520)	0.121	5.805 (1.840–18.309)	0.003 *
PRKCA	1.174 (0.785–1.757)	0.434	1.639 (1.142–2.353)	0.007 *
RAC1	0.119 (0.006–2.322)	0.160	0.535 (0.022–13.040)	0.701
TRPM1	1.439 (0.358–5.778)	0.608	7.869 (1.464–42.286)	0.016 *
Total	0.333 (0.001–0.041)	<0.001 *	0.329 (0.196–0.551)	<0.001 *

Abbreviations: ATG12, autophagy-related 12 gene; BCL11B, BAF chromatin remodeling complex subunit BCL11B; CACNA1C, calcium voltage-gated channel subunit alpha1 C gene; CCKAR, cholecystokinin A receptor gene; NR3C1, nuclear receptor subfamily 3 group C member 1 gene; PRKCA, protein kinase C α gene; RAC1, Rac family small GTPase 1 gene; TRPM1, transient receptor potential cation channel subfamily M member 1 gene; Total: PRS for the whole SNPs; OR: odds ratio; CI: confidence interval; PRS: polygenic risk scores; MCI: mild cognitive impairment. * Represents the significance of gene score association analysis (*p* < 0.05).

**Table 5:** Interaction effect analysis of the association between PRS and bone mineral density on MCI.

Gene	BMD × PRS ^1^			BMD × PRS ^2^		
	OR	95%CI	*p*-Value	OR	95%CI	*p*-Value
ATG12	0.917	0.601–1.398	0.686	0.858	0.551–1.336	0.497
BCL11B	1.003	0.855–1.178	0.964	1.010	0.847–1.203	0.915
CACNA1C	1.124	0.652–1.938	0.675	1.148	0.636–2.072	0.647
CCKAR	0.917	0.310–2.710	0.875	0.594	0.184–1.922	0.384
NR3C1	1.804	0.685–4.750	0.232	1.852	0.625–5.491	0.267
PRKCA	0.638	0.463–0.879	0.006 *	0.593	0.418–0.841	0.003 *
RAC1	0.304	0.025–3.686	0.349	0.348	0.023–5.199	0.444
TRPM1	1.025	0.274–3.835	0.971	0.932	0.227–3.833	0.922
Total	0.900	0.589–1.376	0.626	0.962	0.603–1.535	0.872

Abbreviations: ATG12, autophagy-related 12 gene; BCL11B, BAF chromatin remodeling complex subunit BCL11B; CACNA1C, calcium voltage-gated channel subunit alpha1 C gene; CCKAR, cholecystokinin A receptor gene; NR3C1, nuclear receptor subfamily 3 group C member 1 gene; PRKCA, protein kinase C α gene; RAC1, Rac family small GTPase 1 gene; TRPM1, transient receptor potential cation channel subfamily M member 1 gene; Total: PRS for the whole SNPs; OR: odds ratio; CI: confidence interval; BMD: bone mineral density; PRS: polygenic risk scores; MCI: mild cognitive impairment. * Represents the significance of gene score association analysis (*p* < 0.05). ^1^ Crude results. ^2^ Results adjust for age, gender, education, smoking status, and alcohol drinking status.

## Data Availability

The raw data supporting the conclusions of this article will be made available by the authors upon reasonable request, further inquiries can be directed to the corresponding author.

## Supplementary Materials

The following supporting information can be downloaded at: https://www.mdpi.com/article/10.3390/genes14040828/s1, Table S1: SNP information for this study.

## Abbreviations

AD, Alzheimer’s disease; BMD, Bone mineral density; CHB, Chinese Han Beijing; CI, Confidence interval; DNA, Deoxyribonucleic acid; DXA, Dual-energy X-ray absorptiometry; GWAS, Genome-wide association study; MAF, Minor allele frequency; MCI, Mild cognitive impairment; MICE, Multiple imputation by chained equations; MOCA, Montreal Cognitive Assessment; OR, Odds ratios; PCR, Polymerase chain reaction; PRS, Polygenic risk scores; SD, Standard deviation; SNPs, Single nucleotide polymorphisms.

## References

[1] Vega J.N., Newhouse P.A. (2014). Mild cognitive impairment: Diagnosis, longitudinal course, and emerging treatments. Curr. Psychiatry Rep..

[2] Petersen R.C., Lopez O., Armstrong M.J., Getchius T.S.D., Ganguli M., Gloss D., Gronseth G.S., Marson D., Pringsheim T., Day G.S. (2018). Practice guideline update summary: Mild cognitive impairment: Report of the Guideline Development, Dissemination, and Implementation Subcommittee of the American Academy of Neurology. Neurology.

[3] Gavelin H.M., Dong C., Minkov R., Bahar-Fuchs A., Ellis K.A., Lautenschlager N.T., Mellow M.L., Wade A.T., Smith A.E., Finke C. (2021). Combined physical and cognitive training for older adults with and without cognitive impairment: A systematic review and network meta-analysis of randomized controlled trials. Ageing Res. Rev..

[4] Jia L., Du Y., Chu L., Zhang Z., Li F., Lyu D., Li Y., Li Y., Zhu M., Jiao H. (2020). Prevalence, risk factors, and management of dementia and mild cognitive impairment in adults aged 60 years or older in China: A cross-sectional study. Lancet Public Health.

[5] Lissek V., Suchan B. (2021). Preventing dementia? Interventional approaches in mild cognitive impairment. Neurosci. Biobehav. Rev..

[6] Pan G., King A., Wu F., Simpson-Yap S., Woodhouse A., Phipps A., Vickers J.C. (2021). The potential roles of genetic factors in predicting ageing-related cognitive change and Alzheimer’s disease. Ageing Res. Rev..

[7] Trampush J.W., Yang M.L., Yu J., Knowles E., Davies G., Liewald D.C., Starr J.M., Djurovic S., Melle I., Sundet K. (2017). GWAS meta-analysis reveals novel loci and genetic correlates for general cognitive function: A report from the COGENT consortium. Mol. Psychiatry.

[8] Ohi K., Sumiyoshi C., Fujino H., Yasuda Y., Yamamori H., Fujimoto M., Shiino T., Sumiyoshi T., Hashimoto R. (2018). Genetic Overlap between General Cognitive Function and Schizophrenia: A Review of Cognitive GWASs. Int. J. Mol. Sci..

[9] Xu C., Zhang D., Wu Y., Tian X., Pang Z., Li S., Tan Q. (2017). A genome-wide association study of cognitive function in Chinese adult twins. Biogerontology.

[10] Berridge M.J. (2014). Calcium signalling and psychiatric disease: Bipolar disorder and schizophrenia. Cell Tissue Res..

[11] Sushma, Mondal A.C. (2019). Role of GPCR signaling and calcium dysregulation in Alzheimer’s disease. Mol. Cell Neurosci..

[12] Heck A., Fastenrath M., Coynel D., Auschra B., Bickel H., Freytag V., Gschwind L., Hartmann F., Jessen F., Kaduszkiewicz H. (2015). Genetic Analysis of Association Between Calcium Signaling and Hippocampal Activation, Memory Performance in the Young and Old, and Risk for Sporadic Alzheimer Disease. JAMA Psychiatry.

[13] Kumar A. (2020). Calcium Signaling During Brain Aging and Its Influence on the Hippocampal Synaptic Plasticity. Adv. Exp. Med. Biol..

[14] Khachaturian Z.S., Alzheimer’s Association Calcium Hypothesis Workgroup (2017). Calcium Hypothesis of Alzheimer’s disease and brain aging: A framework for integrating new evidence into a comprehensive theory of pathogenesis. Alzheimers Dement..

[15] Burgess S., Thompson S.G. (2013). Use of allele scores as instrumental variables for Mendelian randomization. Int. J. Epidemiol..

[16] Burgess S., Dudbridge F., Thompson S.G. (2016). Combining information on multiple instrumental variables in Mendelian randomization: Comparison of allele score and summarized data methods. Stat. Med..

[17] Carvalho A., Rea I.M., Parimon T., Cusack B.J. (2014). Physical activity and cognitive function in individuals over 60 years of age: A systematic review. Clin. Interv. Aging.

[18] Pérez-Sousa M., Del Pozo-Cruz J., Olivares P.R., Cano-Gutiérrez C.A., Izquierdo M., Ramírez-Vélez R. (2021). Role for Physical Fitness in the Association between Age and Cognitive Function in Older Adults: A Mediation Analysis of the SABE Colombia Study. Int. J. Environ. Res. Public. Health.

[19] Sewell K.R., Erickson K.I., Rainey-Smith S.R., Peiffer J.J., Sohrabi H.R., Brown B.M. (2021). Relationships between physical activity, sleep and cognitive function: A narrative review. Neurosci. Biobehav. Rev..

[20] McHugh Power J., Tang J., Kenny R.A., Lawlor B.A., Kee F. (2020). Mediating the relationship between loneliness and cognitive function: The role of depressive and anxiety symptoms. Aging Ment. Health.

[21] Li W., Sun H., Xu W., Ma W., Yuan X., Wu H., Kou C. (2021). Leisure activity and cognitive function among Chinese old adults: The multiple mediation effect of anxiety and loneliness. J. Affect. Disord..

[22] Valenzuela M.J., Sachdev P. (2006). Brain reserve and dementia: A systematic review. Psychol. Med..

[23] Killin L.O., Starr J.M., Shiue I.J., Russ T.C. (2016). Environmental risk factors for dementia: A systematic review. BMC Geriatr..

[24] Zhou R., Deng J., Zhang M., Zhou H.D., Wang Y.J. (2011). Association between bone mineral density and the risk of Alzheimer’s disease. J. Alzheimers Dis..

[25] Laudisio A., Fontana D.O., Rivera C., Ruggiero C., Bandinelli S., Gemma A., Ferrucci L., Antonelli Incalzi R. (2016). Bone Mineral Density and Cognitive Decline in Elderly Women: Results from the InCHIANTI Study. Calcif. Tissue Int..

[26] Bendayan R., Kuh D., Cooper R., Muthuri S., Muniz-Terrera G., Adams J., Ward K., Richards M. (2017). Associations of Childhood and Adulthood Cognition with Bone Mineral Density in Later Adulthood: A Population-Based Longitudinal Study. Front. Aging Neurosci..

[27] Kang H.G., Park H.Y., Ryu H.U., Suk S.H. (2018). Bone mineral loss and cognitive impairment: The PRESENT project. Medicine.

[28] Nasreddine Z.S., Phillips N.A., Bédirian V., Charbonneau S., Whitehead V., Collin I., Cummings J.L., Chertkow H. (2005). The Montreal Cognitive Assessment, MoCA: A brief screening tool for mild cognitive impairment. J. Am. Geriatr. Soc..

[29] Kanis J.A. (2002). Diagnosis of osteoporosis and assessment of fracture risk. Lancet.

[30] Jorgensen T.J., Ruczinski I., Kessing B., Smith M.W., Shugart Y.Y., Alberg A.J. (2009). Hypothesis-driven candidate gene association studies: Practical design and analytical considerations. Am. J. Epidemiol..

[31] Singer J.B. (2009). Candidate gene association analysis. Methods Mol. Biol..

[32] Jansen J.H., van der Reijden B.A. (2006). Isolation of RNA and DNA from leukocytes and cDNA synthesis. Methods Mol. Med..

[33] White I.R., Royston P., Wood A.M. (2011). Multiple imputation using chained equations: Issues and guidance for practice. Stat. Med..

[34] Lin X., Song K., Lim N., Yuan X., Johnson T., Abderrahmani A., Vollenweider P., Stirnadel H., Sundseth S.S., Lai E. (2009). Risk prediction of prevalent diabetes in a Swiss population using a weighted genetic score—The CoLaus Study. Diabetologia.

[35] Barral S., Habeck C., Gazes E., De Jager P.L., Bennett D.A., Stern Y. (2017). A Dopamine Receptor genetic variant enhances perceptual speed in cognitive healthy subjects. Alzheimers Dement..

[36] Zheng C., Fu Q., Shen Y., Xu Q. (2012). Investigation of allelic heterogeneity of the CCK-A receptor gene in paranoid schizophrenia. Am. J. Med. Genet. B Neuropsychiatr. Genet..

[37] Porcelli S., Lee S.J., Han C., Patkar A.A., Serretti A., Pae C.U. (2015). CACNA1C gene and schizophrenia: A case-control and pharmacogenetic study. Psychiatr. Genet..

[38] Khalil M., Hollander P., Raucher-Chéné D., Lepage M., Lavigne K.M. (2022). Structural brain correlates of cognitive function in schizophrenia: A meta-analysis. Neurosci. Biobehav. Rev..

[39] Plieger T., Felten A., Splittgerber H., Duke É., Reuter M. (2018). The role of genetic variation in the glucocorticoid receptor (NR3C1) and mineralocorticoid receptor (NR3C2) in the association between cortisol response and cognition under acute stress. Psychoneuroendocrinology.

[40] De Kloet E.R., Joëls M., Holsboer F. (2005). Stress and the brain: From adaptation to disease. Nat. Rev. Neurosci..

[41] McNerney M.W., Sheng T., Nechvatal J.M., Lee A.G., Lyons D.M., Soman S., Liao C.P., O’Hara R., Hallmayer J., Taylor J. (2018). Integration of neural and epigenetic contributions to posttraumatic stress symptoms: The role of hippocampal volume and glucocorticoid receptor gene methylation. PLoS ONE.

[42] Keller J., Gomez R., Williams G., Lembke A., Lazzeroni L., Murphy G.M., Schatzberg A.F. (2017). HPA axis in major depression: Cortisol, clinical symptomatology and genetic variation predict cognition. Mol. Psychiatry.

[43] Kang J.-H. (2014). Protein kinase C (PKC) isozymes and cancer. New J. Sci..

[44] Domínguez-García S., Gómez-Oliva R., Geribaldi-Doldán N., Hierro-Bujalance C., Sendra M., Ruiz F.A., Carrascal L., Macías-Sánchez A.J., Verástegui C., Hernández-Galán R. (2021). Effects of classical PKC activation on hippocampal neurogenesis and cognitive performance: Mechanism of action. Neuropsychopharmacology.

[45] Choi D.S., Wang D., Yu G.Q., Zhu G., Kharazia V.N., Paredes J.P., Chang W.S., Deitchman J.K., Mucke L., Messing R.O. (2006). PKCepsilon increases endothelin converting enzyme activity and reduces amyloid plaque pathology in transgenic mice. Proc. Natl. Acad. Sci. USA.

[46] Jablensky A., Morar B., Wiltshire S., Carter K., Dragovic M., Badcock J.C., Chandler D., Peters K., Kalaydjieva L. (2011). Polymorphisms associated with normal memory variation also affect memory impairment in schizophrenia. Genes. Brain Behav..

[47] Wilker S., Elbert T., Kolassa I.T. (2014). The downside of strong emotional memories: How human memory-related genes influence the risk for posttraumatic stress disorder—A selective review. Neurobiol. Learn. Mem..

[48] Lepichon J.B., Bittel D.C., Graf W.D., Yu S. (2010). A 15q13.3 homozygous microdeletion associated with a severe neurodevelopmental disorder suggests putative functions of the TRPM1, CHRNA7, and other homozygously deleted genes. Am. J. Med. Genet. A.

[49] Hori T., Ikuta S., Hattori S., Takao K., Miyakawa T., Koike C. (2021). Mice with mutations in Trpm1, a gene in the locus of 15q13.3 microdeletion syndrome, display pronounced hyperactivity and decreased anxiety-like behavior. Mol. Brain.

[50] Ershler W.B. (1993). Interleukin-6: A Cytokine for Gerontolgists. J. Am. Geriatr. Soc..

[51] Beek K.J., Rusman T., van der Weijden M.A.C., Lems W.F., van Denderen J.C., Konsta M., Visman I., Nurmohamed M.T., van der Horst-Bruinsma I.E. (2019). Long-Term Treatment With TNF-α Inhibitors Improves Bone Mineral Density But Not Vertebral Fracture Progression in Ankylosing Spondylitis. J. Bone Min. Res..

[52] Lopez J.R., Lyckman A., Oddo S., Laferla F.M., Querfurth H.W., Shtifman A. (2008). Increased intraneuronal resting [Ca^2+^] in adult Alzheimer’s disease mice. J. Neurochem..

[53] Khrimian L., Obri A., Ramos-Brossier M., Rousseaud A., Moriceau S., Nicot A.S., Mera P., Kosmidis S., Karnavas T., Saudou F. (2017). Gpr158 mediates osteocalcin’s regulation of cognition. J. Exp. Med..

[54] Rossi M., Battafarano G., Pepe J., Minisola S., Del Fattore A. (2019). The Endocrine Function of Osteocalcin Regulated by Bone Resorption: A Lesson from Reduced and Increased Bone Mass Diseases. Int. J. Mol. Sci..

[55] Funk J.L., Mortel K.F., Meyer J.S. (1991). Effects of estrogen replacement therapy on cerebral perfusion and cognition among postmenopausal women. Dement. Geriatr. Cogn. Disord..

