# Supplementary material for: The Association of Calcium Signaling Pathway Gene Variants, Bone Mineral Density and Mild Cognitive Impairment in Elderly People
Source: Genes (Basel). 2023 Mar 30;14(4):828. doi: 10.3390/genes14040828 (PMC10137633; doi:10.3390/genes14040828)
Supplement: Supplementary file 1 [file genes-14-00828-s001.zip › genes-2268260-supplementary.pdf]

Supplementary Table S1 SNP information for this study.

Gene	SNP	Chr: position	Variant type	Alleles(major/minor)
ATG12	rs1058600	chr5:115831016	Non Coding Transcript Variant	C/T
ATG12	rs26537	chr5:115841317	Intron Variant	T/C
BCL11B	rs1152781	chr14:99172933	3 Prime UTR Variant	T/G
BCL11B	rs1152783	chr14:99176023	Synonymous Variant	C/G
CACNA1C	rs1006737	chr12:2236129	Intron Variant	G/A
CACNA1C	rs1051375	chr12:2679713	Synonymous Variant	G/A
CACNA1C	rs215976	chr12:2585472	Missense Variant	C/T
CACNA1C	rs216008	chr12:2611971	Synonymous Variant	C/T
CACNA1C	rs2470425	chr12:1968731	2KB Upstream Variant	C/G
CACNA1C	rs58619945	chr12:2051659	Intron Variant	G/A
CACNA1C	rs723672	chr12:2052395	Intron Variant	T/C
CACNA1C	rs4765868	chr12:1970159	5 Prime UTR Variant	C/G
CCKAR	rs17846984	chr4:26481586	3 Prime UTR Variant	C/T
CCKAR	rs1800857	chr4:26489489	Intron Variant	A/G
CCKAR	rs1800908	chr4:26490600	2KB Upstream Variant	C/A
CCKAR	rs2725301	chr4:26483007	Intron Variant	A/G
CCKAR	rs2854038	chr4:26491587	2KB Upstream Variant	A/G
CCKAR	rs6448456	chr4:26490352	5 Prime UTR Variant	G/C
CCKAR	rs1799723	chr4:26490553	2KB Upstream Variant	T/C
CCKAR	rs1800855	chr4:26489495	Intron Variant	T/A
NR3C1	rs10052957	chr5:143407136	Intron Variant	G/A
NR3C1	rs12655166	chr5:143429707	Intron Variant	T/C
NR3C1	rs258751	chr5:143282715	Missense Variant	G/A
NR3C1	rs33388	chr5:143317730	Intron Variant	T/A
NR3C1	rs41400245	chr5:143436433	2KB Upstream Variant	C/T
NR3C1	rs41423247	chr5:143399010	Intron Variant	G/C
NR3C1	rs61757411	chr5:143437459	2KB Upstream Variant	G/T
NR3C1	rs6191	chr5:143278591	Non Coding Transcript Variant	A/C
NR3C1	rs6196	chr5:143281925	Synonymous Variant	A/G
NR3C1	rs6877893	chr5:143347628	Intron Variant	A/G
PRKCA	rs1005651	chr17:66441893	Intron Variant	A/C
PRKCA	rs11079657	chr17:66435948	Intron Variant	G/A
PRKCA	rs2227857	chr17:66688960	Synonymous Variant	G/A
PRKCA	rs2228945	chr17:66788904	Synonymous Variant	G/A
PRKCA	rs2286674	chr17:66804434	3 Prime UTR Variant	A/G
PRKCA	rs228883	chr17:66447877	Intron Variant	C/T
PRKCA	rs61687889	chr17:66756394	Intron Variant	A/G

PRKCA	rs7220996	chr17:66808582	3 Prime UTR Variant	G/A
PRKCA	rs7342847	chr17:66806426	3 Prime UTR Variant	T/C
PRKCA	rs7342969	chr17:66806071	3 Prime UTR Variant	A/G
PRKCA	rs9909004	chr17:66310015	Intron Variant	C/T
PRKCA	rs3764401	chr17:66301711	2KB Upstream Variant	T/C
PRKCA	rs4790904	chr17:66783036	Intron Variant	T/C
PRKCA	rs8464	chr17:66810598	3 Prime UTR Variant	C/A
RAC1	rs10951982	chr7:6382925	Intron Variant	G/A
RAC1	rs12536544	chr7:6396270	Intron Variant	A/G
RAC1	rs12977	chr7:6402802	3 Prime UTR Variant	C/T
RAC1	rs33999234	chr7:6372756	2KB Upstream Variant	A/G
RAC1	rs4720672	chr7:6404208	500B Downstream Variant	T/C
RAC1	rs702482	chr7:6380568	Intron Variant	A/T
RAC1	rs836478	chr7:6392059	Intron Variant	T/C
RAC1	rs9374	chr7:6402740	3 Prime UTR Variant	G/A
TRPM1	rs11070718	chr15:31001251	3 Prime UTR Variant	C/T
TRPM1	rs11070811	chr15:31101879	Intron Variant	C/T
TRPM1	rs2241493	chr15:31070149	Missense Variant	T/C
TRPM1	rs28441327	chr15:31103143	Intron Variant	C/T
TRPM1	rs3784588	chr15:31002451	Missense Variant	A/G
TRPM1	rs3809578	chr15:31102334	Intron Variant	C/T
